# Administration of Dietary Microalgae Ameliorates Intestinal Parameters, Improves Body Weight, and Reduces Thawing Loss of Fillets in Broiler Chickens: A Pilot Study

**DOI:** 10.3390/ani11123601

**Published:** 2021-12-19

**Authors:** Miroslava Anna Šefcová, Francisco Santacruz, César Marcelo Larrea-Álvarez, Christian Vinueza-Burgos, David Ortega-Paredes, Gabriel Molina-Cuasapaz, Jessica Rodríguez, William Calero-Cáceres, Viera Revajová, Esteban Fernández-Moreira, Marco Larrea-Álvarez

**Affiliations:** 1 Research Unit, Life Science Initiative (LSI), Quito 170102, Ecuador; miroslava.sefcova@gmail.com (M.A.Š.); cmla88@hotmail.com (C.M.L.-Á.); 2 School of Biological Science and Engineering, Yachay-Tech University, Hacienda San José, Urcuquí 100650, Ecuador; francisco.santacruz@yachaytech.edu.ec; 3 Unidad de Investigación de Enfermedades Transmitidas por Alimentos y Resistencia a los Antimicrobianos (UNIETAR), Facultad de Medicina Veterinaria y Zootecnia, Universidad Central del Ecuador, Quito 170129, Ecuador; cvinueza@uce.edu.ec (C.V.-B.); daortegap@gmail.com (D.O.-P.); 4 Facultad de Ciencias Médicas Enrique Ortega Moreira, Carrera de Medicina, Universidad Espíritu Santo, Samborondón 0901952, Ecuador; 5 Facultad de Ciencias Agropecuarias y Recursos Naturales, Carrera de Medicina Veterinaria, Universidad Técnica de Cotopaxi, Latacunga 050101, Ecuador; edie.molina7278@utc.edu.ec (G.M.-C.); jessica.rodriguez0606@utc.edu.ec (J.R.); 6 UTA-RAM-One Health, Department of Food and Biotechnology Science and Engineering, Universidad Técnica de Ambato, Ambato 180207, Ecuador; wr.calero@uta.edu.ec; 7 Department of Morphological Disciplines, University of Veterinary Medicine and Pharmacy, 040 01 Košice, Slovakia; viera.revajova@uvlf.sk

**Keywords:** body weight, broiler chickens, goblet cells, intestinal morphometry, microalgae, *Porphyridium cruentum*, *Tetraselmis chuii*, thawing loss, *Tysochrysis lutea*

## Abstract

**Simple Summary:**

Microalgae synthesize a wide variety of bioactive compounds, such as polysaccharides, PUFAs, and pigments. In addition, microalgae are known for positively influencing growth and intestinal parameters in birds and thus have been contemplated as potential feed additives. Nevertheless, several species widely used in aquaculture have not received much attention in terrestrial animal nutrition. Therefore, in this investigation, we used biomass from *Tysochrysis lutea*, *Tetraselmis chuii*, and *Porphyridium cruentum* to evaluate its safety and usefulness for improving intestinal architecture, body weight, and selected meat quality parameters. Dietary administration of the different biomasses showed positive effects regarding intestinal architecture, which were associated with the observed body weight. Furthermore, the thawing weight loss of fillets decreased after supplementation with *T. chuii*. Hence, these outcomes suggest that dietary microalgae might be considered as a promising bio-friendly alternative for feed additive production.

**Abstract:**

This pilot investigation aimed at studying the feasibility of using a low dose (0.2%) of dietary microalgae as a means of improving intestinal morphometry, body weight, and selected meat quality parameters in broilers. A total of 72 one-day-old ROSS 308 male chicks were randomly separated into four groups; three experimental pens in which the birds were fed with biomass from *Tysochrysis lutea*, *Tetraselmis chuii*, and *Porphyridium cruentum* over 30 days and a control group. *T. chuii* and *P. cruentum* had a positive effect with regard to body weight. In treated animals, duodenal and ileal sections showed characteristic tall and thin villi, with serrated surfaces and goblet cell differentiation. In both sections, values of the villus-height-to-crypt-depth ratio were increased by microalgae ingestion. The thawing weight loss of fillets was reduced in *T. chuii*-fed animals. The positive effects exerted by *T. chuii* and *P. cruentum* on intestinal architecture were associated with the improved body weight. Arguably, these outcomes exhibit the potential of using these species to enhance growth performance in broiler chickens by promoting gut homeostasis and thus nutrient absorption.

## 1. Introduction

In animal husbandry, antibiotics are principally used to prevent, control, and treat diseases. However, they have also been extensively used as growth promoters [1,2]; this practice has been banned in Europe and the U.S. [3,4] but is still widespread in other regions, especially in rural areas, where farmers tend to depend more on antibiotics. Various countries lack legislative measurements to control antibiotic use. Moreover, farmers are not appropriately trained due to the lack of educational programs. Countries with deficient and ill-administrated health systems will suffer the greatest problems when coping with antibiotic resistance [5,6]. Diverse alternatives for promoting animal growth and well-being have been studied, especially in poultry production. For instance, probiotics are known for enhancing growth performance and immune parameters [7,8,9,10,11]. Likewise, extracts from various plants have proved useful for such purposes [12,13,14]. Algae administration has also yielded positive outcomes with regard to animal performance and immunity [15,16].

Algae constitute a heterogeneous group of photosynthetic organisms present in a variety of sizes, shapes, and colors (which include red, green, and golden brown). They are divided into two principal groups: macroalgae, often referred to as seaweeds, and microalgae [17]. Algae have been incorporated into fish feed in farms and hatcheries. In particular, microalgae are considered important as they synthesize valuable compounds, such as polyunsaturated fatty acids, polyphenols, essential amino acids and proteins, pigments, and various polysaccharides [18]. In fact, microalgae-enriched feed has proved useful for promoting growth performance in broiler chickens before [19,20,21,22]. Nonetheless, most experiments have made use of *Chlorella* sp. and *Arthospira* (ex. *Spirulina*) [18,23]. Effects of other, less common species have also been reported, including *Schizochytrium* and *Amphora* sp. [24,25]. In addition, the effects of microalgae administration on intestinal morphometry have not been extensively documented. One study showed that feed enriched with 2.5% *Chlorella* improves intestinal integrity by positively influencing villus height and crypt depth [17].

Various species produced for aquaculture have yet to be tested as components of poultry feed. In golden-brown microalgae, *Tysochrysis lutea* belongs to a group of auspicious marine haptophytes with high lipid content and the production of polyunsaturated fatty acids. Moreover, this species is a rich source of the pigment fucoxanthin, which has nutraceutical and pharmaceutical applications [26]. In green algae, *Tetraselmis chuii* is known for its straightforward cultivation and high nutritional value. This microalga accumulates high quantities of important compounds, such as pigments, proteins, and polyunsaturated fatty acids [27]. Among red microalgae, *Porphyridium cruentum* is considered a promising organism for the food industry because it can synthesize and secrete high amounts of sulfated polysaccharides as well as lipids and polyunsaturated fatty acids [28], which are recognized as important antioxidant, anti-inflammatory, and cytotoxic agents [29]. Consequently, in this pilot study, we aimed at evaluating the effects of dietary microalgae mainly on intestinal morphometry but also on body weight and selected meat quality parameters in broiler chickens, using biomass from species traditionally used in aquaculture: *T. lutea*, *T. chuii*, and *P. cruentum*.

## 2. Materials and Methods

The study was carried out in the Experimental Centre for Animal Research of the Veterinary Medicine Faculty, Central University, Ecuador. The facility is located in the parish of Uyumbicho, situated 23 km southeast of Quito. Experiments were conducted following the guidelines for poultry management provided by the Agency for the Regulation and Control of Phytosanitary and Animal Health (AGROCALIDAD, technical resolution n. 0017). The Ethics Committee on the Use of Animals in Research and Teaching of the San Francisco de Quito University (USFQ) revised and approved the related protocols (reference number: 2020-008).

### 2.1. Microalgae Biomass

The freeze-dried microalgae powder of *T. lutea*, *T. chuii*, and *P. cruentum* was purchased from Necton S.A., Olhão, Portugal (https://necton.pt/ accessed on 2 June 2021). *Tysochrysis lutea* belongs to the phylum Haptophyta; these organisms are known to contain both chlorophylls *a* and *c*. The presence of the carotenoid accessory pigment fucoxanthin is partly responsible for their often-golden-brown color [26]. This biomass possesses crude protein (40%), crude fat (6%), and crude ash (23%). *T. chuii* belongs to the phylum Chlorophyta; these organisms are characterized by the presence of chlorophylls *a* and *b*, along with the carotenoids violaxanthin, antheraxanthin, zeaxanthin, neoxanthin, and lutein [27]. This biomass possesses crude protein (35%), crude fat (5%), and crude ash (30%). *P.* *cruentum* belongs to the phylum Rhodophyta, which contains chlorophyll *a* as well as α- and β-carotene, lutein, and zeaxanthin. Additionally, these organisms produce water-soluble pigments known as phycobilins [28,29]. This biomass possesses crude protein (35%), crude fat (5%), and crude ash (31%). The components of these microalgae suggest a potentially high nutritional value for birds.

### 2.2. Experimental Setup

A total of 72 one-day-old male broiler chicks (ROSS 308) were weighed (50 ± 5.8 g on average) and then randomly divided into four groups. Each experimental group was set up in an individual pen (3 m × 3 m), divided into nine subgroups (1 m × 1 m). Each subgroup contained two birds; the subgroups were housed separately from the others. For body weight measurements, both animals were weighed and the averaged values were used for analyses. For sampling, one chicken was selected per subgroup (*N* = 9). The subgroup was considered the experimental unit, as each of the screened birds was independently allocated to treatment conditions and experimental intervention and could not influence others on the measured outcome [30]. All chickens were fed basal diets free of probiotics, antibiotics, or anticoccidiostats with nutritional components of broilers for starter (1–11 days) and finisher (12–30 days) diets (Table 1). Feed and water were available ad libitum throughout the entire experiment, which lasted 30 days. Animals were sacrificed at this age as the main objective of the research was to assess the influence of treatments on intestinal morphometry before the onset of critical growth stages (weeks 5–6). This study was not aimed at determining growth and meat quality parameters in detail. Nonetheless, the chicken body weight was registered as a mean to monitor the health of animals during the trial. Moreover, cooking and thawing loss of fillets were also determined. The first group were fed with pelleted basal diet and thus considered the control. Birds from groups 2–4 were fed an enriched diet with freeze-dried microalgae powder. All experimental diets were provided with the same dose of biomass (2g/kg); group 2 (TL) was supplemented with *T. lutea*, group 3 (TC) with *Tetraselmis chuii*, and group 4 (PC) with *Porphyridium cruentum*. Chickens were raised on a floor covered with hardwood shavings. The ambient temperature was maintained between 30 and 32 °C during the first week and gradually decreased by 3 °C every week, i.e., on day 7 (27–29 °C), 14 (24–26 °C), 21 (21–23 °C), and 28 (19–21 °C). The relative humidity was kept between 50 and 60%. During the first seven days, the birds were exposed to a regime of 23 h of light (intensity 30–40 Lux) and 1 h of dark, which was followed by one regime of 21 h of light (intensity 5–10 Lux) and 4 h of dark, until the end of the experiment. Housing and environmental conditions abided by the ROSS Broiler Management Guide [31]. 

### 2.3. Body Weight

All animals were weighed, and the averaged values were used for analyses. This was carried out on day 5 and then on days on which the rearing temperature was modified (7, 14, 21, and 28). Additionally, weights were registered on days 29 and 30.

### 2.4. Morphometrical Analyses

On day 30, all animals were weighed and one bird per subgroup was electrically stunned and euthanized by bleeding. From the intestine, the loop of the duodenum and the tract before the ileocolic junction were collected. Segments were fixed with a 10% formalin solution for 48 h. Then, samples were dehydrated by serial washes using ethyl alcohol (70–100%), diaphanized using xylol, and finally embedded in paraffin blocks. These blocks were sliced in three longitudinal sections of 5 μm thick blades using a rotary microtome (Leica RM2235, Wetzlar and Mannheim, Germany) and stained with hematoxylin and eosin (HE staining). Images were captured and processed using the Motic Images Plus 2.0 (Motic, Hong Kong, China). Villus height and crypt depth were determined per segment on uninjured villi, six at least, this was performed four times for a total of 24 readings per animal. The villus choice was based on the presence of an intact lamina propria. The villus-height-to-crypt-depth ratio was calculated as hitherto described [8]. The number of goblet cells per 100 intestinal epithelial cells in six intact villi was determined using the aforementioned program, as described previously [32].

### 2.5. Meat Quality Parameters

At the end of the experiment, nine animals per group were sampled for their meat quality and six 10 g fillets from the *Pectoralis major* of each euthanized bird were dissected and cleaned. For cooking loss (three fillets), samples were placed in thermotolerant plastic bags and put in a water bath until 70 °C was reached; this was maintained for about 10 min. Samples were cooled on ice to 5 °C and then weighed. For thawing loss (three fillets), breast samples were dried, trimmed, and stored at –18 °C. A week later, thawing of the frozen fillets was carried out at 5 °C for 1 day and then the final weight was determined. The differences between initial and final weights were considered the values of loss. The values of fillets were averaged for statistical analyses. These procedures were performed as previously described [24].

### 2.6. Statistical Analyses

First, Shapiro–Wilk’s test and Levene’s test were used for assessing normality and homogeneity of variance, respectively. For normally distributed and homoscedastic data, a one-way analysis of variance was used for determining significant differences between experimental groups, along with a Tukey post hoc test. For normally distributed and heteroscedastic data, Welch’s ANOVA and Welch’s *t*-test were applied. For non-normally distributed and homoscedastic data, Kruskal–Wallis test and Mann–Whitney U test (Wilcoxon rank sum test) were used. In the latter case, since the distribution is non-symmetrical, the mean is affected as a measure of the central tendency of distribution. Thus, medians were used because they reflect the center of distribution more appropriately in such conditions. Pearson’s *r* correlation coefficient was performed to identify potential relationships between the indicators. Analyses were carried out in MATLAB^®^ version 9.9.9341360 (MathWorks, Natick, MA, USA) (R2016a).

## 3. Results

### 3.1. Effects of Diet on Body Weight

Compared to control conditions, no significant differences regarding body weight were observed during the first two weeks in microalgae-fed groups. From day 28, chickens of the *T. chuii* group were heavier than those fed a basal diet. At the end of the experiment, administration of *T. chuii* and *P. cruentum* proved to significantly increase chicken body weight compared to control conditions, by 16% and 13%, respectively (Table 2). 

### 3.2. Histological Findings and Small Intestine Morphometric Measures

**Figure 1:**
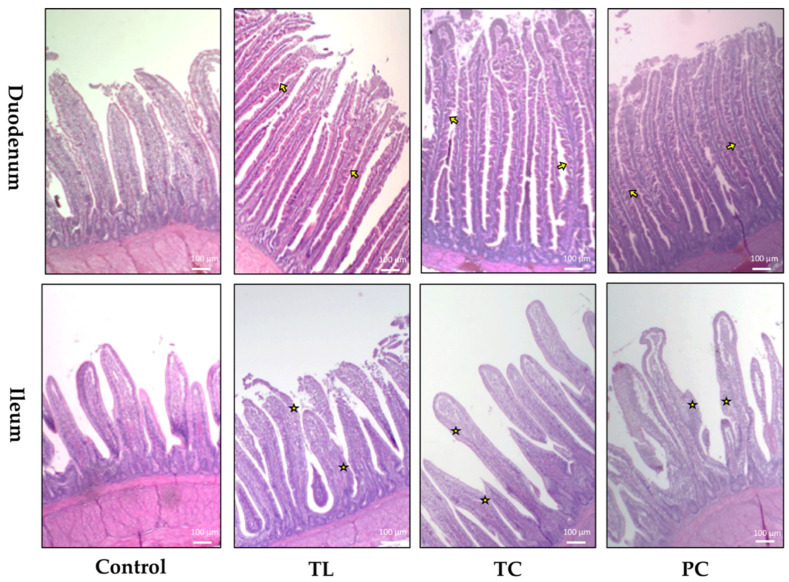
Illustrative photomicrographs showing the effects of dietary microalgae on intestinal morphology in broiler chickens (40× magnification, HE staining). Sections of the duodenum and ileum show thin and tall villi in treated animals, whereas thick and short villi were characteristic of untreated birds. In duodenal sections, marked serrated surfaces were observed in the tall separated villi in chickens supplemented with microalgae biomass (yellow arrows). In ileal sections, intestinal epithelial proliferation and goblet cell differentiation can be observed in microalgae-enriched groups (yellow star). TL: *Tysochrysis lutea*; TC: *Tetraselmis chuii*; PC: *Porphyridium cruentum*. HE: hematoxylin and eosin.

Illustrative micrographs of duodenum and ileum in 40× magnification are shown in Figure 1. Sections of duodenal and ileal segments of untreated birds exhibited short and thick villi with limited crypt development, whereas those of microalgae-fed animals appeared healthier, with tall and thin villi, along with active crypts with increased size and rows of enterocytes. In sections from the duodenum, tall and thin villi displayed serrated surfaces and broad tips related to multiplying enterocytes. In ileal sections, proliferation of the epithelium and goblet cell differentiation could be observed. 

Chickens fed with microalgae biomass displayed an increase in villus height and crypt depth in the duodenum, except for those supplemented with *T. chuii*. No differences were found regarding the villus-height-to-crypt-depth ratio (Table 3). In ileal sections, the height of villi was augmented in all experimental treatments, whereas only *P. cruentum* increased the crypt depth. A higher ratio of villus height to crypt depth was detected in all microalgae-enriched groups (Table 3). Pearson’s *r* coefficient analysis showed a positive correlation between the weight of chickens and the villus-height-to-crypt-depth ratio (0.977; *p* < 0.05). This suggests that the increment in body weight could be associated with healthier intestinal epithelia. In duodenal sections, goblet cells were found in higher numbers in microalgae-enriched groups than in control conditions, whereas in ileal sections, only *P. cruentum* induced a positive effect (Table 3).

### 3.3. Meat Quality Parameters

Cooking loss of the not fully developed pectoral muscles was not altered by microalgae supplementation, but there was improvement in thawing loss observed in animals fed with *T. chuii* compared to the control, TL, and PC groups *(*Table 4).

## 4. Discussion

Microalgae are known to be a reliable source of nutrients and have long been exploited in aquaculture [18]. These organisms have also been integrated into animal nutrition, although to a lesser extent [18,23]. Previous studies have shown that the inclusion of dietary microalgae in feed could improve growth parameters in broiler chickens [20,21,22]. The majority of experiments regarding microalgae have been carried out using *Spirulina platensis* and *Chlorella vulgaris* [18,23], although effects of other, less common species have also been reported, including *Schizochytrium* sp. and *Amphora coffeaformis* [24,25]. Information regarding supplementation with *T. lutea*, *T. chuii*, and *P. cruentum* in broilers is scarce, with only one study demonstrating that dietary supplementation with *Porphyridium* sp. reduces feed intake although it does not affect body weight [33]. Therefore, we wanted to test the safety and effectiveness of these microalgae, commercially produced for aquaculture, in improving intestinal morphometry, body weight, as well as selected meat quality parameters in broiler chickens. 

*T. chuii* and *P. cruentum* are acknowledged for improving growth performance in shrimp and fish [34,35]. The present outcomes reveal that dietary administration of these species of microalgae had an overall positive effect on chicken body weight. For microalgae, published results suggest a biomass incorporation rate of 2%, or between 1 and 5%. However, such rates are not deemed suitable for mass application due to the costs [18]. Here, we used a dietary incorporation rate of 0.2%, as we wanted to test the effects of a lower dosage. Previous data showed an increase in body weight, compared to control conditions, in birds fed with 0.1% *C. vulgaris* (8%) and *A. coffeaformis* (7.4%) [24]. Similarly, dietary administration of 1% *Chlorella* raised the body weight by 3.5% [22]. These values are lower than those found in the present study, where 0.2% of *T. chuii* and *P. cruentum* augmented the body weight by 16% and 13%, respectively. Another study showed that supplementation (7%) with a product derived from *Schizochytrium* increased growth by 22% [36]. Evidently, the quantity of biomass used influences the extent of microalgal effects on growth. *T. lutea* administration has not led to an improvement in weight parameters in fish [37]. Similarly, in the present study, the group where the feed was enriched with *T. lutea* did not show any differences when compared with the control group. Inclusion of microalgae in feed has not always resulted in higher body weight average values in birds [24,25]. In any case, the positive results with regard to growth performance exhibited here were supported by morphometrical analyses of the small intestine. 

Macroalgae-enriched feed has proven useful for ameliorating intestinal architecture in broiler chickens [15,16]. However, the influence of microalgae administration on intestinal health has not been documented extensively. It has been demonstrated that inclusion of *Chlorella* in diets increases villus height and crypt depth in jejunal and ileal sections [38,39]. Another study showed an improvement in these parameters after administration of extracts enriched with *A. platensis* [40]. Our results are in line with the mentioned studies, as all treatments exerted a positive effect on intestinal integrity. In fact, *T. lutea*, *Tetraselmis* sp., and *P. cruentum* are known for improving intestinal morphology and health in fish [35,41]. In duodenal and ileal sections, villi were characteristically taller and thinner in microalgae-treated birds than in untreated animals, whereas crypt depth was only improved in chickens fed with *P. cruentum*. All microalgae species improved values of the villus-height-to-crypt-depth ratio in ileal sections. Deeper crypts have been associated with a swift regeneration of the villi, which increases their overall height [42]. Longer villi along with a higher ratio of villus height to crypt depth are considered crucial indicators of gastrointestinal health, as they are linked to a greater capacity of nutrient absorption [43,44]. Positive morphometry was correlated with an increase in body weight. Various studies have shown that the administration of probiotics ameliorates the integrity of intestinal epithelial cells, which leads to a more efficient absorption of nutrients, which ultimately improves growth performance [7,8,45,46]. The current outcomes demonstrate that dietary supplementation with the tested microalgae was beneficial for an improvement of intestinal epithelial architecture, which could have led to a more proficient intake of food and thus increased body weight. These physiological conditions would allow birds to enhance nutrient absorption during critical stages of growth (weeks 5 and 6).

The goal of this preliminary study was to evaluate the safety and usefulness of using the screened species as feed additives. The biomasses used in this study are known to contain fat, protein, and oligosaccharides (Section 2.1). It has been shown that ω-3 fatty acids are capable of improving intestinal morphology in broilers [47]. *T. chuii* produces hexadecatetraenoic acid (C16:4ω-3), stearidonic acid (C18:4ω-3), and oleic acid (C18:1ω-9), while *P. cruentum* also accumulates ω-3 fatty acids, such as docosahexaenoic acid (C22:ω-6) and eicosapentaenoic acid (C22:ω-5) [47,48]; these compounds are known for their high nutritional value for poultry production [47]. As green algae, *T. chuii* contains xanthophylls such as lutein and zeaxanthin, along with β-carotene [27]; the latter two are known to increase the height of villi in the small intestine of broiler chickens [49]. Plant extracts containing lutein proved to activate intestinal nutrient absorption by increasing the surface of the villi [50]. Similarly, *P. cruentum* produces β-carotene, lutein, and zeaxanthin. These microorganisms accumulate various phycobilins, including phycoerythrin, which is responsible for their red color [28,29]. These compounds are known for exerting growth promoting effects and improving intestinal morphology in broiler chickens [51]. Oligosaccharides are reported to enhance intestinal structures [52]. In particular, *Porphyridium* sp. synthesizes important amounts of structural polysaccharides and exopolysaccharides. These compounds are recognized for inducing morphological modifications in the small intestine as well as augmenting the number of goblet cells in the mucosal layer of rats [53]. However, monogastric animals cannot digest such oligosaccharides, so they reach the colon and are fermented by a diverse group of microorganisms [54]. Microbial fermentation produces short-chain fatty acids (e.g., butyrate) that play an important role in intestinal epithelium development [55]. These are considered important sources of energy and are notorious for stimulating mucus production and secretion [56]. It could be argued that the results observed in this study maybe be related to the aforenoted compounds, which are known for stimulating intestinal and growth parameters. Further work must be emphasized on producing extracts for compound acquisition and concentration.

The oligosaccharide side chains on mucins can interact with bacterial adhesins and therefore prevent their reaching and damaging the epithelium [56]. Goblet cells make part of the epithelial cells lining the villi. These cells synthesize the major component of mucus, mucin 2 (Muc2) [57]. Maintenance of the mucus layer of the intestinal epithelium is crucial for nutrient transport, protection, and lubrication [58]. Plant-derived compounds, probiotics, and prebiotics have proven useful for ameliorating intestinal histology, including goblet cell count, in untreated chickens and in chickens challenged with bacterial pathogens [12,13,59]. Here, we showed that microalgae administration promotes goblet cell differentiation in both duodenum and ileum. This seems relevant in the context of a bacterial infection, since goblet cells and their products make part of the first line of defense against undesired microorganisms. Therefore, these microalgae species could be applied with the aim of reducing the impact of pathogenic bacteria, which has been demonstrated for other algae in the context of a *Salmonella* infection [60,61]. 

Data on meat quality parameters showed that microalgae did not modify cooking loss, but *T. chuii* administration proved to reduce thawing loss compared to control conditions. These results are comparable to those of a previous study showing no differences in cooking loss but an improvement in thawing loss when using a microalgae-derived product [20]. Another report showed that *S. platensis* administration did not alter either parameter [62] whereas dietary (0.1%) *C. vulgaris*, *S. platensis*, and *A. coffeaformis* reduced cooking loss but did not alter thawing loss [24]. Results appear related to the species of microalgae used. In any case, dietary administration does not seem to have a negative impact on the analyzed parameters.

Microalgae represent a novel area of relevance for animal production, especially with regard to nutrition and health. These photosynthetic organisms synthesize and accumulate several important compounds valuable for improving intestinal architecture. This is of prime importance, not only because nutrient absorption efficiency could be enhanced, but also because intestinal epithelium serves as a physical barrier against invasive pathogens. In addition, biotechnology has proved useful for producing important biomolecules by helping genetically engineer the plastid genome of the model alga *Chlamydomonas reinhardtii.* For instance, potential vaccines against common diseases such as the avian influenza virus and infectious bronchitis virus (IBV) have been produced in such a way [63,64]. This opens up the possibility of oral delivery of vaccines by enriching feed with dried microalgae, thereby reducing the requirements for purification and cold-chain transportation [65]. Undoubtedly, microalgae biotechnology will permit the design of bespoke strains with superior productivity that could be applied in animal nutrition.

## 5. Conclusions

The present outcomes suggest that the screened species, especially *T. chuii* and *P. cruentum*, have the potential to be used as feed additives in nutrition. Administration of dietary microalgae (0.2%) proved useful in ameliorating intestinal morphology and epithelial barrier; this was associated with improved body weight. Furthermore, *T. chuii* supplementation reduced thawing loss of dissected fillets. These results provide important insights into the current understanding of microalgae use in poultry production. However, further investigation must be carried out for refining our knowledge of the properties of these microorganisms and assessing the effects not only on animal welfare and nutrition but also on quality products.

## Figures and Tables

**Table 1:** Feed components and proximate composition of starter (1–11 days) and finisher (12–30 days) diets of the control and experimental groups.

Ingredients (%)	Control		Experimental Groups	
	Starter	Finisher	Starter	Finisher
Ground corn	52.99	57.23	52.88	57.12
Soybean meal (CP, 48.0%)	36.3	32.07	36.23	32.01
Calcium carbonate	1.52	1.49	1.52	1.49
Monocalcium phosphate	1.07	0.82	1.07	0.82
Sodium chloride	0.31	0.26	0.31	0.26
Crude (vegetal) fat	7.4	7.71	7.39	7.69
Antimycotic	0.10	0.08	0.10	0.08
Mycotoxin sequestrant	0.05	0.05	0.05	0.05
Antioxidant	0.02	0.02	0.02	0.02
Phytase	0.01	0.01	0.01	0.01
^1^ Vitamin and mineral premix	0.23	0.26	0.23	0.26
Microalgae-derived protein	-	-	0.08	0.08
Microalgae-derived fat	-	-	0.01	0.01
Microalgae-derived crude ash	-	-	0.05	0.05
Microalgae-derived carbohydrate, fiber, rest of biomass	-	-	0.06	0.06
Nutrient specifications				
^2^ ME Kcal/kg diet	3000	3190	3000	3190
Lysine (%)	1.48	1.25	1.48	1.25
Methionine + cysteine (%)	1.07	0.99	1.07	0.99
Methionine (%)	0.59	0.50	0.59	0.50
Threonine (%)	0.97	0.85	0.97	0.85
Valine (%)	1.06	0.97	1.06	0.97
Isoleucine (%)	0.95	0.86	0.95	0.86
Arginine (%)	1.51	1.38	1.51	1.38
Tryptophan (%)	0.24	0.22	0.24	0.22
Leucine (%)	1.58	1.45	1.58	1.45
Crude protein (%)	22.50	21.00	22.50	21.00
Ca (%)	1.00	0.85	1.00	0.85
Available P (%)	0.50	0.40	0.50	0.40
Mg (%)	0.40	0.40	0.40	0.40
Na (%)	0.23	0.16	0.23	0.16
Cl (%)	0.22	0.19	0.22	0.19
K (%)	0.96	0.72	0.96	0.72
Choline (mg/kg)	1700	1600	1700	1600
Linoleic acid (%)	1.25	1.15	1.25	1.15

^1^ Vitamin premix incorporated in each kg of basal diet: vitamin A, 11,000 IU; vitamin D3, 4500 IU; vitamin E, 70 IU; vitamin K, 3.1 mg; vitamin B1, 3.1 mg; vitamin B2, 7.2 mg; vitamin B6, 4.5 mg; vitamin B12, 0.014 mg; biotin, 0.3 mg; pantothenic acid, 16 mg; and folic acid, 1.8 mg. Mineral premix incorporated in each kg of basal diet: Mn, 120 mg; Zn, 110 mg; Fe, 20 mg; Cu, 16 mg; I, 1.25 mg; and Se, 0.30 mg. ^2^ ME, metabolizable energy. TL: *Tysochrysis lutea*; TC: *Tetraselmis chuii*; PC: *Porphyridium cruentum*.

**Table 2:** Effects of microalgae administration on body weight.

	Experimental Groups			
	Control	TL	TC	PC
Body weight (g)				
Day 5	108.67 ± 10.5	111.44 ± 13.3	116.78 ± 16.9	110.44 ± 15.7
Day 7	154.78 ± 16.4	155 ± 18.4	172.11 ± 21	157.67 ± 18.7
Day 14	391 ± 34.2	373.78 ± 38.7	416.67 ± 39.3	395.67 ± 42.3
Day 21	800.67 ± 51.5	760 ± 61.3	891.11 ± 72.9 ^■^	867.33 ± 94.9 ^■^
Day 28	1336 ± 56.7	1316.8 ± 95.8	1530.2 ± 85.3 *^■^	1433.9 ± 111.1 ^■^
Day 29	1398.8 ± 64.8	1399 ± 105.8	1611.4 ±100.3 *^■^	1540.2 ± 125.3 *^■^
Day 30	1425 ± 72.7	1494.6 ± 117	1658.6 ± 103.7 *^■^	1613 ± 146.1 *

Values are means ± SE (*N* = 9). * Designates significant differences from the control group (*p* < 0.05) and ^■^ from the TL group. TL: *Tysochrysis lutea*; TC: *Tetraselmis chuii*; PC: *Porphyridium cruentum*.

**Table 3:** Effects of microalgae biomass administration on morphometric characteristics of the duodenum and ileum in broiler chickens.

Small Intestine Sections	Experimental Groups			
	Control	TL	TC	PC
Duodenum				
Villus height (μm)	2659.06 ± 324.9	3233.92 ± 210.1 *	3454.45 ± 353.9 *	3842.46 ± 243.1 *^■^
Crypt depth (μm) ^1^	357.27(69.5)	404.79(38.6) *	395.32(52.8)	484.90(48.7) *^■▲^
Villus-height-to-crypt-depth ratio	7.48 ± 1.0	8.15 ± 0.9	8.89 ± 1.0 *	8.19 ± 0.9
Goblet cell count	20.82 ± 5.2	31.94 ± 4.9 *	35.65 ± 7.2 *	36.35 ± 4.0 *
Ileum				
Villus height (μm)	1270.70 ± 143.7	1659.30 ± 228.6 *	1981.50 ± 117.4 *^■^	2303.90 ± 180.7 *^■▲^
Crypt depth (μm)	323.92 ± 29.4	362.40 ± 45.4	374.63 ± 54.06	406.94 ± 57.9 *
Villus-height-to-crypt-depth ratio	3.81 ± 0.6	4.17 ± 0.4 *	5.51 ± 0.8 *	5.8 ± 0.8 *^■^
Goblet cell count	41.37 ± 4.5	46.62 ± 9.6	45.55 ± 3.5	52.96 ± 5.6 *

Values are means ± SE (*N* = 9). ^1^ Values are medians plus their corresponding interquartile range (IQR). * Designates significant differences from the control group (*p* < 0.05), ^■^ from the TL group, and ^▲^ from the TC group. TL: *Tysochrysis lutea*; TC: *Tetraselmis chuii*; PC: *Porphyridium cruentum*.

**Table 4:** Effects of microalgae biomass administration on selected meat quality parameters in broiler chickens.

Parameters	Experimental Groups			
	Control	TL	TC	PC
Cooking loss (g)	1.28 ± 0.1	1.30 ± 0.2	1.41 ± 0.2	1.42 ± 0.3
Thawing loss (g)	0.83 ± 0.1	0.71 ± 0.2	0.49 ± 0.1 *^■^	0.89 ± 0.3 ^▲^

Values are means ± SE (*N* = 9). * Designates significant differences from the control group (*p* < 0.05), ^■^ from the TL group, and ^▲^ from the TC group. TL: *Tysochrysis lutea*; TC: *Tetraselmis chuii*; PC: *Porphyridium cruentum*.

## Data Availability

Data supporting these findings are available in Open Science Framework at doi: 10.17605/OSF.IO/PR4TD.
